# Association of Increased MIEN1 Copy Number With Worse Outcomes in Lung Adenocarcinoma and Cervical Cancer

**DOI:** 10.7759/cureus.111880

**Published:** 2026-07-01

**Authors:** Sami Soliman, Teresa Thomas, Vayda Barker, Michael Aboujaoude, George Angelakakis, Toriana Dabkowski, George Blanck

**Affiliations:** 1 Molecular Medicine, University of South Florida Morsani College of Medicine, Tampa, USA; 2 College of Medicine, University of South Florida (USF) Health, Tampa, USA

**Keywords:** cervical cancer, copy number variation, erbb2, lung adenocarcinoma, mien1, outcomes

## Abstract

Lung adenocarcinoma is the most common subtype of lung cancer and a significant contributor to cancer mortality globally. This has driven the development of targeted therapies, particularly those aimed at genetic alterations in certain genes, such as EGFR and ALK. ERBB2 (HER2) has also emerged as a potential oncogenic driver and therapeutic target in lung adenocarcinoma. Notably, ERBB2 is in close proximity on chromosome 17 to GRB7 and MIEN1, which are potential contributors to invasion and metastasis. Using TCGA-LUAD (The Cancer Genome Atlas Lung Adenocarcinoma) and CPTAC-3 (Phase III of the Clinical Proteomic Tumor Analysis Consortium) lung adenocarcinoma datasets, copy number variations (CNVs) for GRB7, ERBB2, and MIEN1 and their associations with various survival parameters were obtained. Results indicated that increased copy number (CN) of MIEN1 was significantly associated with worse disease-free survival (DFS) in both TCGA-LUAD and CPTAC-3 lung adenocarcinoma datasets and was significantly associated with worse progression-free survival (PFS) in the TCGA-LUAD dataset. There is also evidence showing a similar relationship in cervical cancer, which also has established links to ERBB2. CNV analyses in the TCGA-CESC and CGCI-HTMCP-CC (Cancer Genome Characterization Initiative-HIV+ Tumor Molecular Characterization Project-Cervical Cancer) cervical cancer datasets revealed that only increased CN of MIEN1 was statistically significantly associated with worse overall survival (OS) in both datasets. These CNV analyses suggest that MIEN1 should be further investigated as a potential contributor to oncogenesis.

## Introduction

As of 2020, lung cancer was the second most commonly diagnosed cancer and the leading cause of cancer deaths, accounting for 18% of all cancer-related deaths [1]. Lung adenocarcinoma was the most common type of lung cancer diagnosed in both men and women [2]. The subtypes of lung adenocarcinoma can vary in five-year survival rates [3], and distinct treatments exist for these subtypes. Thus, treatments for lung adenocarcinoma often focus on driver mutations or gene fusions, such as mutated EGFR (epidermal growth factor receptor) or ALK-EML4 (anaplastic lymphoma kinase-echinoderm microtubule-associated protein-like 4 fusion) [4]. This tailored approach enhances the efficacy of lung adenocarcinoma treatment.

Another example of a genomic alteration that defines a lung adenocarcinoma subtype and dictates specific treatments is mutated or amplified ERBB2 (HER2; human epidermal growth factor receptor 2). One study found that ERBB2 was amplified in 14.3% of adenocarcinomas and that increased ERBB2 gene amplification was associated with a worse prognosis [5]. However, it was noted that there was often overlap between ERBB2 gene amplification and other driver mutations, e.g., mutations of EGFR and KRAS (Kirsten rat sarcoma virus oncogene homolog) [5]. Nevertheless, another study determined that high levels of ERBB2 gene amplification led to a rare but clinicopathologically distinct subgroup of non-small cell lung cancer (NSCLC) [6]. While implicated in cancer, this suggests some uncertainty regarding the exact role of ERBB2 in oncogenic activity. Data on treatment efficacy are also mixed. Trastuzumab, which targets the ERBB2 protein, has shown limited efficacy in vitro and in vivo for lung adenocarcinoma; however, the modified trastuzumab deruxtecan has been shown to inhibit lung adenocarcinoma growth and induce apoptosis [6]. Further clarification of the exact role of ERBB2 in NSCLC and lung adenocarcinoma, therefore, remains an important goal.

The genes GRB7 (growth factor receptor-bound protein 7) and MIEN1 (migration and invasion enhancer 1) are located close to ERBB2 on chromosome 17 (genome.ucsc.edu). GRB7 is an adapter protein that binds several receptor tyrosine kinases, including EGFR and ERBB2, and is often upregulated in cancers [7]. When GRB7 is co-expressed with EGFR or ERBB2, there is a potential increase in metastasis [7]. MIEN1 is a regulator of migration and invasion and is often overexpressed in cancers, including NSCLC [8]. One study found that MIEN1 protein upregulation, due to decreased E3 ubiquitin ligase synoviolin, promotes NSCLC metastasis [9]. Additionally, suppression of miR-26b, an inhibitor of MIEN1 mRNA translation, in NSCLC leads to increased migration and invasion [10]. Given the oncogenic potential of GRB7 and MIEN1 and their genomic proximity to ERBB2, the role of ERBB2 amplification in NSCLC requires further consideration of the possible contributions of these proximal genes.

ERBB2 has also been indicated as a potential target for the treatment of gynecological malignancies, including cervical cancer. A study reported that among 228 cervical cancer samples, 17% had amplification of ERBB2 [11], and therapy intended to negate ERBB2 protein function in vitro successfully inhibited the growth of cervical cancer cell lines with ERBB2 mutations [12]. Some research on GRB7 has suggested that GRB7 overexpression can lead to inhibition of apoptosis and increased invasion in cervical cancer [13]. However, there is minimal research on the relationship between MIEN1 and cervical cancer.

In sum, MIEN1 and GRB7, given their chromosomal proximity to ERBB2, are important to consider as potential drivers of lung adenocarcinoma and cervical cancer. To explore this hypothesis, we evaluated copy number (CN) amplifications of GRB7, ERBB2, and MIEN1 in lung adenocarcinoma and cervical cancer datasets to determine whether increased copy number variations (CNVs) were associated with distinct survival outcomes. We employed a previously benchmarked, precision-guided CNV approach [14,15] with the potential to associate outcomes with amplifications of specific genes. Subsequently, multivariate analyses were conducted on selected data.

## Materials and methods

Access to the genomics file datasets

The Genomic Data Commons (GDC) (https://portal.gdc.cancer.gov/) was used to access the manifest and sample sheet for the cancer datasets employed in this study. The Cancer Genome Atlas Lung Adenocarcinoma (TCGA-LUAD, phs000178) and Clinical Proteomic Tumor Analysis Consortium (CPTAC-3 lung adenocarcinoma, phs001287) datasets were accessed by filtering for "bronchus and lung" and "adenomas and adenocarcinomas." Whole exome sequencing (WXS) data in BAM (Binary Alignment/Map) format were selected. The files were accessed according to the National Institutes of Health (NIH) database of Genotypes and Phenotypes (dbGaP) project approval numbers 6300 and 31752, respectively. The Cancer Genome Atlas Cervical Squamous Cell Carcinoma and Endocervical Adenocarcinoma Collection (TCGA-CESC, phs000178) dataset was selected and filtered to the primary site, cervix uteri. Whole genome sequencing (WGS) and WXS data in BAM format were selected and accessed via NIH dbGaP approval number 6300. Finally, the Cancer Genome Characterization Initiative: HIV+ Tumor Molecular Characterization Project-Cervical Cancer (CGCI-HTMCP-CC, phs000235) dataset was selected and filtered to the primary site, cervix uteri. WGS data in BAM format were selected and accessed via NIH dbGaP approval number 20312. The link to the downloaded manifests for all of the above is provided in the Appendices (Parts S1-S5).

CNV assessments

CNVs were assessed using the original code benchmarked in references [14-16]. The chromosomal locations of the genes studied in this report, needed for the CNV assessment algorithm, were obtained from the human genome browser (https://genome.ucsc.edu/) (GRCh38/hg38). The chromosomal locations were as follows: GRB7 (chr17:39737938-39747284), ERBB2 (chr17:39688094-39728657), and MIEN1 (chr17:39728510-39730532). For the WGS and WXS datasets obtained and analyzed, ratios of tumor to blood sequencing reads were calculated for the genes indicated above to represent CNVs, as described in references [14-16] (Tables S6-S20). The code used for this process, specifically finalized for this study, is publicly accessible at https://github.com/ss4coding/CNV/tree/main/cnv.

Survival analyses

Clinical datasets were accessed via https://www.cbioportal.org/, with reference to the following lung adenocarcinoma dataset subcategory identifiers: TCGA, PanCancer Atlas; CPTAC-3 lung adenocarcinoma, GDC. For all analyses, the cases were subdivided into groups based on the ratio of tumor to blood sequencing read counts for the indicated genes (Results). Cases in the TCGA-LUAD, CPTAC-3 lung adenocarcinoma, and TCGA-CESC WXS datasets were grouped by a tumor-to-blood read ratio of 1.5 or greater versus less than 1.5. TCGA-CESC cases, based on WGS read counts, were grouped according to the upper and lower 50th percentiles of the read count ratios. For the CGCI-HTMCP-CC dataset, the survival data ("demographic.days_to_death") were accessed from the GDC (https://portal.gdc.cancer.gov/). The dataset was filtered using the same parameters detailed for the survival analysis of CGCI-HTMCP-CC, and the "clinical" file was accessed (Appendices, Part 21). Days were converted to and approximated as months by dividing by 30. The cases were grouped according to the upper and lower 50th percentiles of the tumor-to-blood sequencing read ratios (Appendices, Parts 22-24). Case ID groups were analyzed via a Kaplan-Meier (KM) assessment using a publicly available script derived from survminer ggsurvplot (https://github.com/ss4coding/CNV/tree/main/km). Note that, for this report, all Python scripts were run using Python 3.6.2 (Python Software Foundation, Beaverton, Oregon). Single time point (STP) analysis was conducted by obtaining the proportion alive at an STP along with the sample size for input into MedCalc's publicly available comparison of proportions calculator (https://www.medcalc.org/calc/comparison_of_proportions.php).

Gene expression analyses

The RNAseq values (RSEM, RNAseq by expectation-maximization) for the TCGA-LUAD (using identifiers: PanCancer Atlas; "Batch normalized from Illumina HiSeq_RNASeqV2") and CPTAC-3 lung adenocarcinoma (using identifiers: CPTAC-3 lung adenocarcinoma, GDC; "mRNA expression") datasets were obtained from cbioportal.org (Appendices, Parts 25 and 26). For both the TCGA-LUAD and CPTAC-3 lung adenocarcinoma datasets, the cases identified from the CNV assessments and RNAseq data extraction were matched by case ID. A Pearson correlation test (https://www.medcalc.org/calc/test_correlation.php) was conducted, and the relevant statistical values are reported (Results). Two-sample Student's t-tests (https://www.medcalc.org/calc/comparison_of_means.php), with associated box-and-whisker plots, were created for distinct CNV case ID groupings based on CN ratios of 1.5 or greater versus less than 1.5 (Results).

Multivariate analysis

The TCGA-LUAD PanCancer dataset was accessed via https://www.cbioportal.org/. For the CGCI-HTMCP-CC dataset, the clinical data were accessed via https://portal.gdc.cancer.gov/. Multivariate analyses were conducted as described by Thomas et al. [17].

## Results

MIEN1 gene CNs and survival

**Figure 1:**
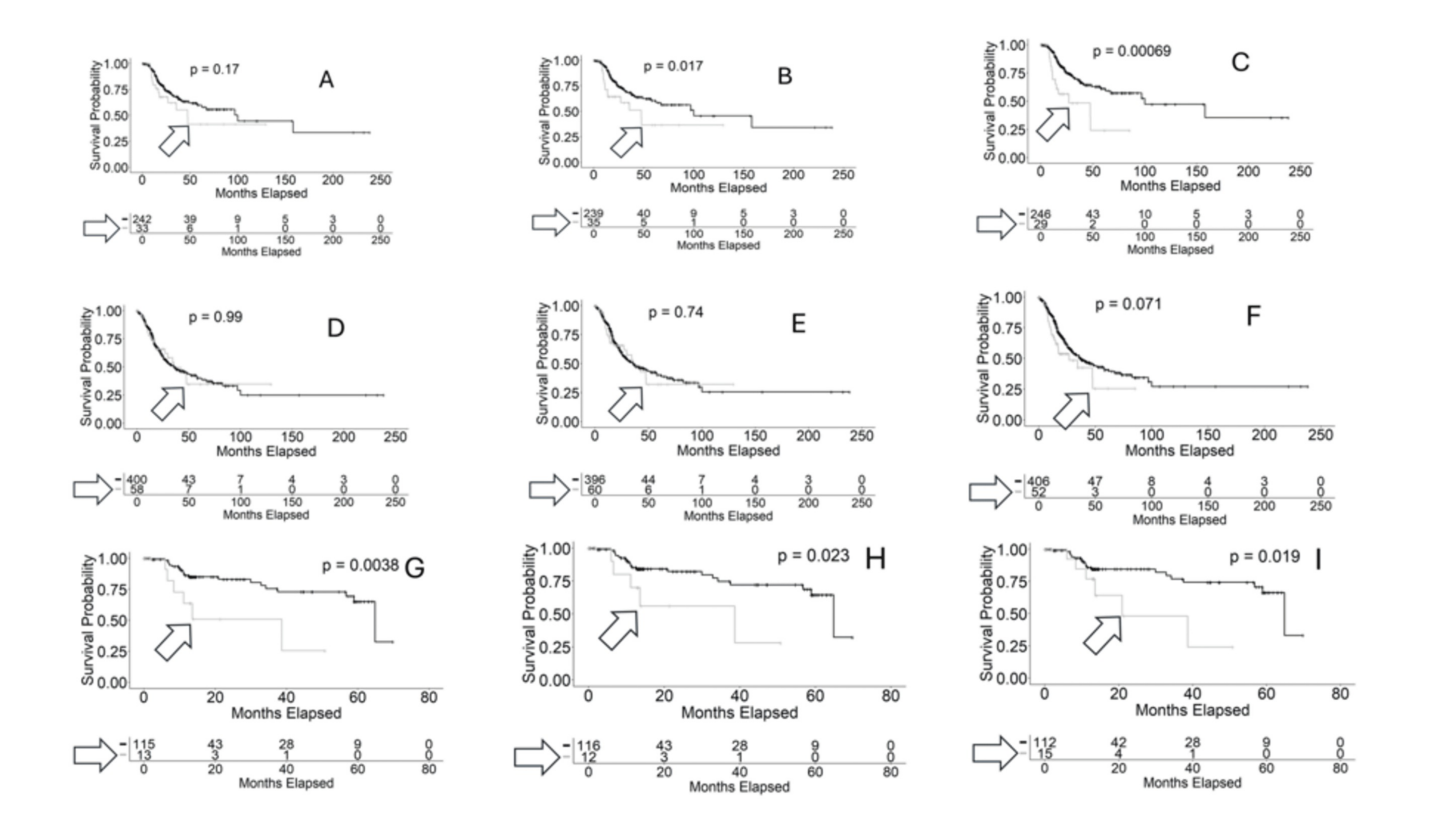
KM analyses of lung adenocarcinoma cases based on copy number (CN) levels of GRB7, ERBB2, and MIEN1. The gray line (arrowhead) indicates a tumor-to-blood sequencing read ratio of 1.5 or greater; the black line indicates a ratio of less than 1.5. (A) Tumor-to-blood sequencing read ratio of 1.5 or greater vs. less than 1.5 for GRB7, disease-free survival (DFS), TCGA-LUAD. (B) Tumor-to-blood sequencing read ratio of 1.5 or greater vs. less than 1.5 for ERBB2, DFS, TCGA-LUAD. (C) Tumor-to-blood sequencing read ratio of 1.5 or greater vs. less than 1.5 for MIEN1, DFS, TCGA-LUAD. (D) Tumor-to-blood sequencing read ratio of 1.5 or greater vs. less than 1.5 for GRB7, progression-free survival (PFS), TCGA-LUAD. (E) Tumor-to-blood sequencing read ratio of 1.5 or greater vs. less than 1.5 for ERBB2, PFS, TCGA-LUAD. (F) Tumor-to-blood sequencing read ratio of 1.5 or greater vs. less than 1.5 for MIEN1, PFS, TCGA-LUAD. (G) Tumor-to-blood sequencing read ratio of 1.5 or greater vs. less than 1.5 for GRB7, DFS, CPTAC-3 lung adenocarcinoma. (H) Tumor-to-blood sequencing read ratio of 1.5 or greater vs. less than 1.5 for ERBB2, DFS, CPTAC-3 lung adenocarcinoma. (I) Tumor-to-blood sequencing read ratio of 1.5 or greater vs. less than 1.5 for MIEN1, DFS, CPTAC-3 lung adenocarcinoma. KM: Kaplan-Meier, CN: copy number, DFS: disease-free survival, TCGA-LUAD: The Cancer Genome Atlas Lung Adenocarcinoma, PFS: progression-free survival, CPTAC-3: Clinical Proteomic Tumor Analysis Consortium 3.

To determine whether MIEN1 CNs were associated with survival differences in cases of lung adenocarcinoma (TCGA-LUAD and CPTAC-3 lung adenocarcinoma), KM analyses were performed to assess two case groups, i.e., cases with greater CNs compared with cases with lower CNs (Figure 1). The TCGA-LUAD set of cases was divided into groups with sequencing read ratios of 1.5 or greater versus those with a ratio of less than 1.5 for GRB7, ERBB2, and MIEN1 (Methods), i.e., MIEN1 and two genes adjacent to MIEN1 (Introduction). For disease-free survival (DFS), the genes GRB7 (Figure 1A; logrank p = 0.17), ERBB2 (Figure 1B; logrank p = 0.017), and MIEN1 (Figure 1C; logrank p = 0.00069) were evaluated. ERBB2 and MIEN1 demonstrated strong distinctions in DFS probability, with cases having greater CNs of MIEN1 demonstrating worse DFS probability. For PFS, the genes GRB7 (Figure 1D; logrank p = 0.99), ERBB2 (Figure 1E; logrank p = 0.74), and MIEN1 (Figure 1F; logrank p = 0.071) were evaluated. None of the genes demonstrated statistically significant differences based on the log-rank p-values. However, an STP analysis representing proportional PFS differences for cases with different MIEN1 CNs was performed. At 51.55 months, cases with lower MIEN1 CNs had a PFS of 43.06%, whereas cases with higher CNs had a PFS of 25.49% (STP p = 0.0154). There were no statistically significant differences at any STP for PFS between the different CN groups for the GRB7 or ERBB2 genes.

The above subdivision of cases (Methods) was applied to the CPTAC-3 lung adenocarcinoma dataset, and cases with greater CNs were compared with those with lower CNs. For DFS, the genes GRB7 (Figure 1G; logrank p = 0.0038), ERBB2 (Figure 1H; logrank p = 0.023), and MIEN1 (Figure 1I; logrank p = 0.019) were evaluated. In all cases, those with greater CNs for their respective genes had worse DFS probabilities. 

Evaluation of TCGA-CESC cases

**Figure 2:**
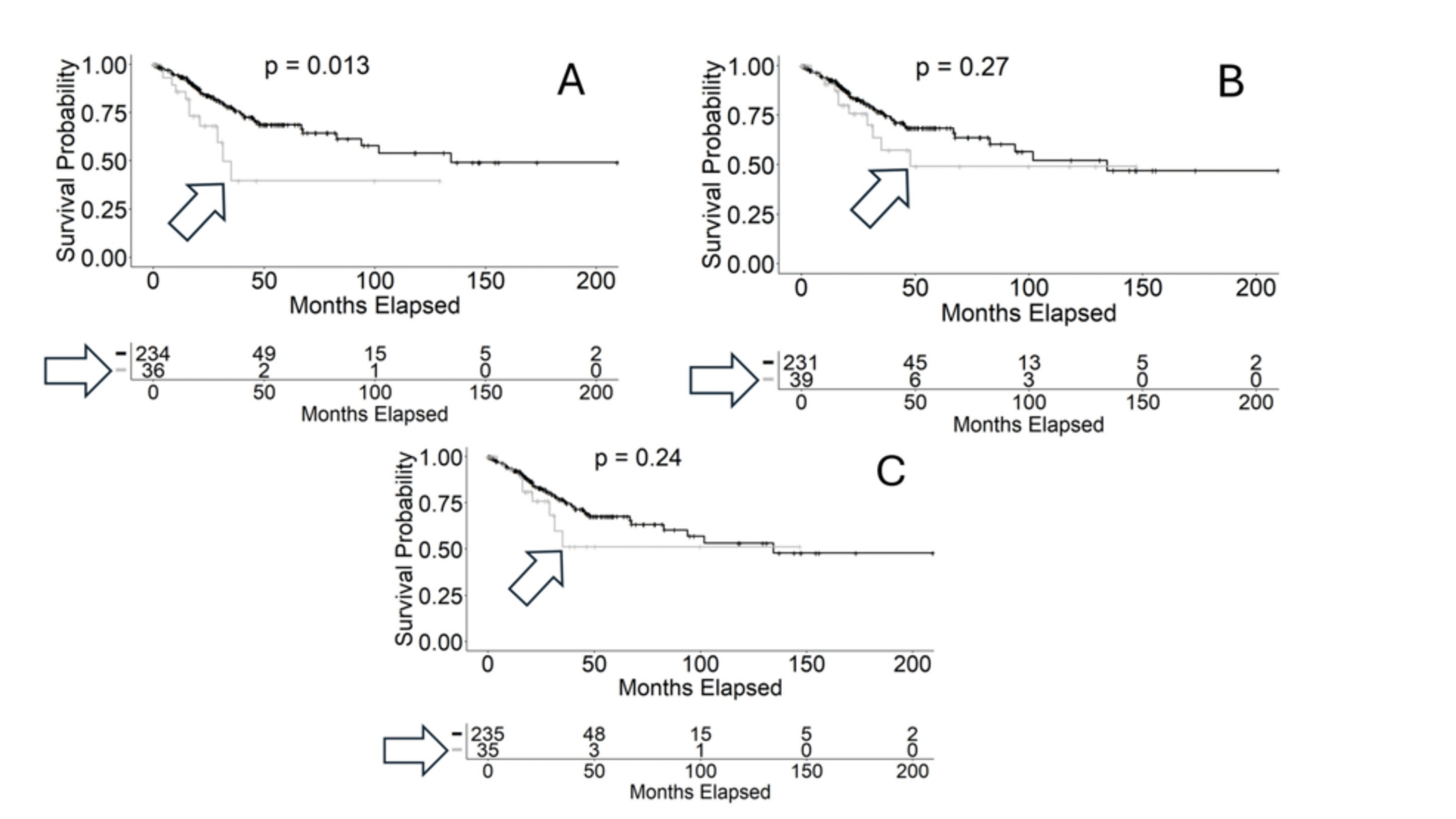
KM analyses of OS in TCGA-CESC cases based on CNs of GRB7, ERBB2, and MIEN1, based on analyses of WXS files. The gray line (arrowhead) indicates a tumor-to-blood sequencing read ratio of 1.5 or greater; the black line indicates a ratio of less than 1.5. (A) Tumor-to-blood sequencing read ratio of 1.5 or greater vs. less than 1.5 for GRB7. (B) Tumor-to-blood sequencing read ratio of 1.5 or greater vs. less than 1.5 for ERBB2. (C) Tumor-to-blood sequencing read ratio of 1.5 or greater vs. less than 1.5 for MIEN1. KM: Kaplan-Meier, OS: overall survival, TCGA-CESC: The Cancer Genome Atlas Cervical Squamous Cell Carcinoma and Endocervical Adenocarcinoma, CNs: copy numbers, WXS: whole-exome sequencing.

The data from TCGA-CESC WXS files were divided into groups based on sequencing read ratios of 1.5 or greater versus those with a ratio of less than 1.5 for GRB7, ERBB2, and MIEN1 (Methods). For the OS parameter, the genes GRB7 (Figure 2A; logrank p = 0.013), ERBB2 (Figure 2B; logrank p = 0.27), and MIEN1 (Figure 2C; logrank p = 0.24) were evaluated. Only the cases represented by GRB7 demonstrated statistically significant differences in OS probabilities, with cases having higher CNs for GRB7 demonstrating worse OS probabilities. STP analyses representing proportional OS differences for cases with different ERBB2 and MIEN1 CNs were performed. At 50 months, cases with lower ERBB2 CNs had an OS of 68.42%, whereas cases with higher CNs had an OS of 49.06% (STP p = 0.0188). At 40 months, cases with lower MIEN1 CNs had an OS of 72.52%, whereas cases with higher CNs had an OS of 51.19% (STP p = 0.0105). 

**Figure 3:**
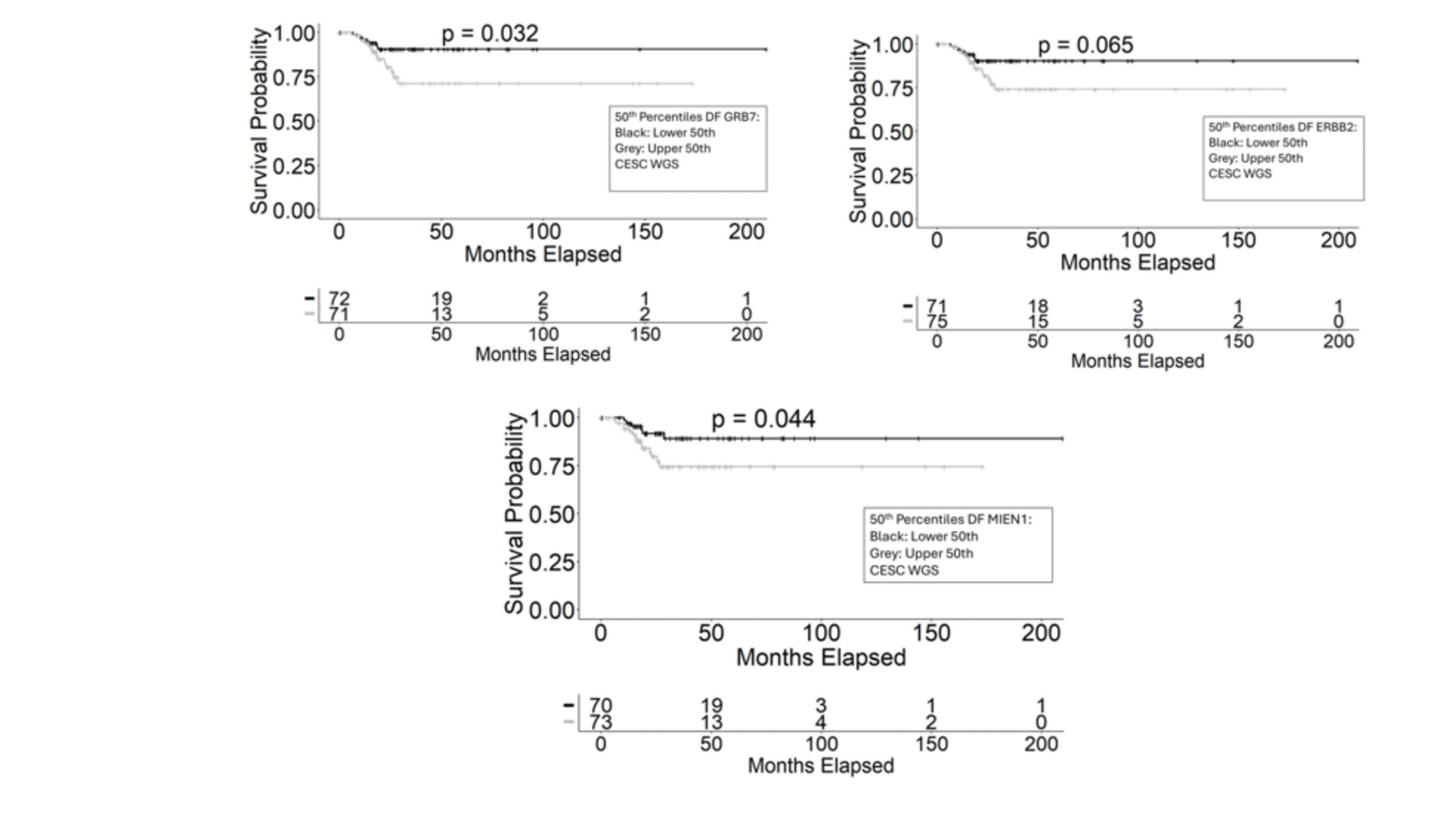
Kaplan-Meier analyses of TCGA-CESC WGS cases indicating differential survival based on amplification levels of GRB7, ERBB2, and MIEN1. The gray line indicates the upper 50th percentile; the black line indicates the lower 50th percentile. (A) Upper 50th percentile tumor-to-blood sequencing read ratio vs. lower 50th percentile for GRB7, DFS, TCGA-CESC WGS. (B) Upper 50th percentile tumor-to-blood sequencing read ratio vs. lower 50th percentile for ERBB2, DFS, TCGA-CESC WGS. (C) Upper 50th percentile tumor-to-blood sequencing read ratio vs. lower 50th percentile for MIEN1, DFS, TCGA-CESC WGS. KM: Kaplan-Meier, TCGA-CESC: The Cancer Genome Atlas Cervical Squamous Cell Carcinoma and Endocervical Adenocarcinoma, WGS: whole-genome sequencing, DFS: disease-free survival.

With TCGA-CESC, WGS data groups of cases were established representing the upper and lower 50th percentile CNs for GRB7, ERBB2, and MIEN1 (Methods). For DFS, the genes GRB7 (logrank p = 0.032), ERBB2 (logrank p = 0.065), and MIEN1 (logrank p = 0.044) were evaluated (Table 1, Figure 3A-C). STP analysis for ERBB2 showed that, at 40 months, cases with lower ERBB2 CNs had a DFS of 90.36%, whereas cases with higher CNs had a DFS of 74.09% (STP p = 0.0154). For all three genes, cases with greater CNs of their respective genes demonstrated worse DFS probabilities. 

**Table 1:** Log-rank p-values for a KM assessment of DFS in TCGA-CESC cases representing the upper and lower 50th percentile CNs, as determined by analyses of the WGS files (Methods). *Indicates statistical significance at p < 0.05. KM: Kaplan-Meier, DFS: disease-free survival, TCGA-CESC: The Cancer Genome Atlas Cervical Squamous Cell Carcinoma and Endocervical Adenocarcinoma, CNs: copy numbers, WGS: whole-genome sequencing.

GRB7	ERBB2	MIEN1
0.032*	0.065	0.044*

Evaluation of CGCI-HTMCP-CC cases

**Figure 4:**
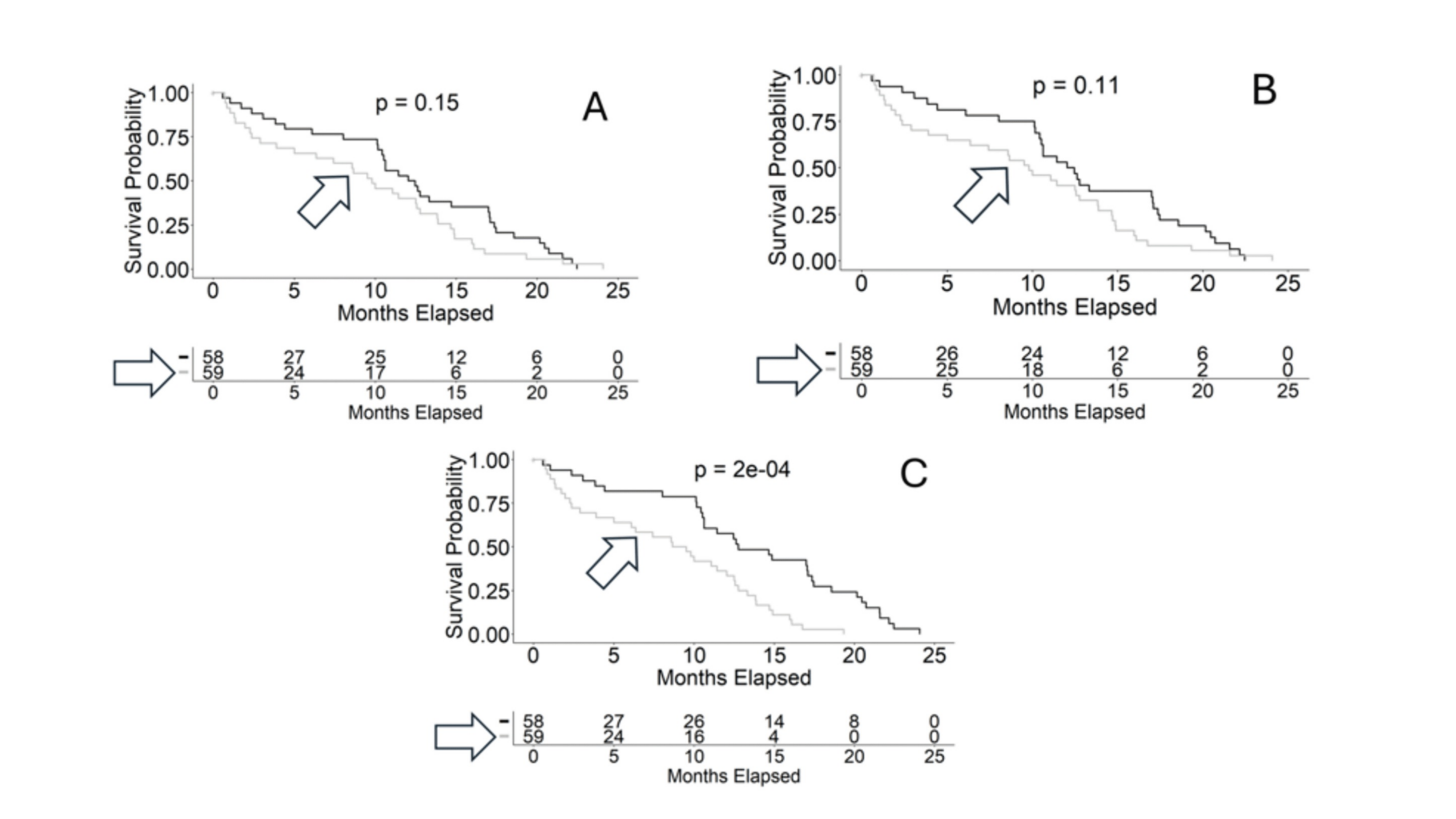
KM analyses of OS in CGCI-HTMCP-CC cases based on CNs of GRB7, ERBB2, and MIEN1, based on analyses of WGS files. The gray line (arrowhead) indicates the upper 50th percentile tumor-to-blood sequencing read ratio; the black line indicates the lower 50th percentile. (A) Upper 50th percentile tumor-to-blood sequencing read ratio vs. lower 50th percentile for GRB7. (B) Upper 50th percentile tumor-to-blood sequencing read ratio vs. lower 50th percentile for ERBB2. (C) Upper 50th percentile tumor-to-blood sequencing read ratio vs. lower 50th percentile for MIEN1. KM: Kaplan-Meier, OS: overall survival, CGCI-HTMCP-CC: Cancer Genome Characterization Initiative–HIV+ Tumor Molecular Characterization Project–Cervical Cancer, CNs: copy numbers, WGS: whole-genome sequencing.

The CGCI-HTMCP-CC data were subdivided based on the 50th percentile standard indicated above for GRB7, ERBB2, and MIEN1. For OS, the genes GRB7 (Figure 4A; logrank p = 0.15), ERBB2 (Figure 4B; logrank p = 0.11), and MIEN1 (Figure 4C; logrank p = 0.0002) were evaluated. Only the analysis representing MIEN1 showed statistically significantly lower OS with greater CNs. 

Correlation of lung adenocarcinoma MIEN1 CNs with RNAseq values

**Figure 5:**
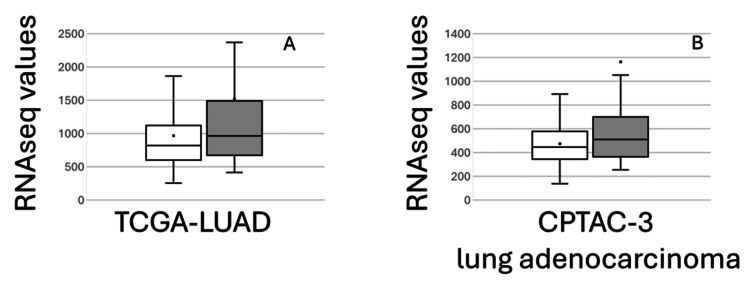
Box-and-whisker plots displaying RNAseq values for MIEN1 grouped by MIEN1 CN levels. Gray indicates a tumor-to-blood sequencing read ratio of 1.5 or greater; white indicates a ratio of less than 1.5. (A) Tumor-to-blood sequencing read ratio of 1.5 or greater vs. less than 1.5, TCGA-LUAD. (B) Tumor-to-blood sequencing read ratio of 1.5 or greater vs. less than 1.5, CPTAC-3 lung adenocarcinoma. RNAseq: RNA sequencing, CN: copy number, TCGA-LUAD: The Cancer Genome Atlas Lung Adenocarcinoma, CPTAC-3: Clinical Proteomic Tumor Analysis Consortium 3.

To determine whether lung adenocarcinoma cases with greater CNs of MIEN1 were associated with increased RNA levels, a Pearson correlation assessment of MIEN1 CNs and RNA levels (Table 2) and a two-sample Student's t-test for case groupings based on MIEN1 CNs and the associated MIEN1 RNAseq values (Figure 5 and Table 3) were performed. The results indicated a statistically significant Pearson correlation between MIEN1 CNs and MIEN1 RNAseq values for the TCGA-LUAD (Pearson p < 0.0001) and CPTAC-3 lung adenocarcinoma (Pearson p < 0.0001) datasets (Table 2). For the two-sample Student's t-test, as in the KM analyses, the TCGA-LUAD and CPTAC-3 lung adenocarcinoma sets of cases were divided into groups with CN ratios of 1.5 or greater versus cases with ratios less than 1.5 (Methods). For TCGA-LUAD, there was a significantly greater mean RNAseq value for cases with increased CNs of MIEN1 (Figure 5A, Table 3; t-test p = 0.0001). For CPTAC-3 lung adenocarcinoma, there was a significantly greater mean RNAseq value for cases with increased CNs of MIEN1 (Figure 5B, Table 3; t-test p = 0.0001). 

**Table 2:** P-values representing the Pearson correlation between MIEN1 RNAseq values and MIEN1 CNs in two lung adenocarcinoma datasets. *Indicates statistical significance at p < 0.05. TCGA-LUAD: The Cancer Genome Atlas Lung Adenocarcinoma, CPTAC-3: Clinical Proteomic Tumor Analysis Consortium 3, RNAseq: RNA sequencing, CNs: copy numbers, R: correlation coefficient, N: sample size.

TCGA-LUAD (Pancancer Atlas)			CPTAC-3 lung adenocarcinoma (GDC)		
N	R	p-value	N	R	p-value
463	0.69627	<0.0001*	170	0.931504	<0.0001*

**Table 3:** Comparison of mean RNAseq values for lung adenocarcinoma cases grouped by MIEN1 tumor-to-blood sequencing read ratios. *Indicates statistical significance at p < 0.05. TCGA-LUAD: The Cancer Genome Atlas Lung Adenocarcinoma, CPTAC-3: Clinical Proteomic Tumor Analysis Consortium 3, RNAseq: RNA sequencing, STDV: standard deviation, N: sample size.

Variables	Ratio less than 1.5			Ratio greater than or equal to 1.5			p-value
	N	Mean	STDV	N	Mean	STDV	
TCGA-LUAD	410	966.883	566.976	53	1518.04	2317.21	0.0001*
CPTAC-3 lung adenocarcinoma	150	474.7	176.2	20	1163.5	2086.9	0.0001*

Multivariate analyses

Multivariate analyses were performed to assess other clinical variables that may correlate with DFS for TCGA-LUAD cases or with OS for CGCI-HTMCP-CC cases. For the former dataset, groups were established representing either a tumor-to-blood MIEN1 sequencing read ratio of 1.5 or greater versus a ratio of less than 1.5, with the results indicating a significant association between higher MIEN1 CNs and worse DFS (HR 2.82, CI 1.36-5.83, p = 0.011) (Table 4). For the latter dataset, the results indicated a significant association between the upper 50th percentile of tumor-to-blood MIEN1 sequencing read ratios and worse OS (HR 3.32, CI 1.46-7.58, p = 0.003) (Table 5). 

**Table 4:** Multivariate analysis of DFS in TCGA-LUAD cases with distinct MIEN1 CNs. *Indicates statistical significance at p < 0.05. HR: hazard ratio, CI: confidence interval, DFS: disease-free survival, TCGA-LUAD: The Cancer Genome Atlas Lung Adenocarcinoma, CNs: copy numbers, CNV: copy number variation, MSI: microsatellite instability.

Characteristics	HR	95% CI	p-value
Diagnosis age	1	0.98-1.02	0.96
CNV ratios (based on ratios)			0.011*
Ratio less than 1.5 (reference value)	—	—	
Ratio 1.5 and greater	2.82	1.36-5.83	
MSIsensor score	1.95	1.20-3.16	0.034*
Radiation therapy			0.5
No (reference value)	—	—	
Yes	1.2	0.71-2.04	
Prior diagnosis			0.37
No (reference value)	—	—	
Yes	1.34	0.72-2.49	
Neoplasm cancer status			<0.001*
Tumor free (reference value)	—	—	
With tumor	11.9	6.19-22.8	
New neoplasm event post initial therapy			0.17
No (reference value)	—	—	
Yes	1.5	0.82-2.72	
Sex			0.61
Female (reference value)	—	—	
Male	1.12	0.73-1.70	

**Table 5:** Multivariate analysis of OS in CGCI-HTMCP-CC cases with MIEN1 CNs. *Indicates statistical significance at p < 0.05. HR: hazard ratio, CI: confidence interval, OS: overall survival, CGCI-HTMCP-CC: Cancer Genome Characterization Initiative–HIV+ Tumor Molecular Characterization Project–Cervical Cancer, CNs: copy numbers, CNV: copy number variation.

Characteristics	HR	95% CI	p-value
Age	1	0.96-1.03	0.78
CNV ratio (based on percentiles)			0.003*
Lower 50th percentile (reference value)	—	—	
Upper 50th percentile	3.32	1.46-7.58	
Clinical diagnosis (m)			0.1
M0 (reference value)	—	—	
M1	6.53	0.31-139	
MX	0.31	0.04-2.51	
Clinical diagnosis (n)			0.5
N0 (reference value)	—	—	
N1	1.97	0.36-10.6	
NX	3.3	0.38-28.5	
Treatment or therapy			0.33
No (reference value)	—	—	
Yes	0.69	0.32-1.45	

## Discussion

Principal findings

Results reported here are consistent with the association of CN variations of GRB7, ERBB2, and MIEN1 with survival probabilities in both lung adenocarcinoma and cervical cancer datasets. For TCGA-LUAD and CPTAC-3 lung adenocarcinoma, MIEN1-based KM analyses indicated a statistically significant association with worse DFS probabilities. Also, higher CNs of MIEN1 were associated with significantly increased MIEN1 RNA expression in both the TCGA-LUAD and CPTAC-3 lung adenocarcinoma datasets. Assessments of the cervical cancer datasets, TCGA-CESC and CGCI-HTMCP-CC, indicated similar results. For the TCGA-CESC WXS and WGS datasets, increased CNs of each of the three genes were significantly related to worse OS and DFS probabilities, respectively. In analyses of the CNs of GRB7, ERBB2, and MIEN1 for the CGCI-HTMCP-CC dataset, increased CNs of only MIEN1 were related to significantly worse OS outcomes. In sum, across both lung adenocarcinoma and cervical cancer, increased CNs of the MIEN1 gene were most consistently related to worse outcomes.

Results in the context of what is known and clinical implications

The above findings are consistent with the previously established roles of GRB7, ERBB2, and MIEN1 in oncogenesis in lung adenocarcinoma [7-10]. However, the close proximity of GRB7, ERBB2, and MIEN1 on chromosome 17 may complicate attempts to achieve a precise understanding of the role of the amplification and increased expression of each of these genes in tumor development. Specifically, while ERBB2 has received significant attention as a therapeutic target in both lung adenocarcinoma [5,6,11,12] and cervical cancer [13,18], the results reported here are consistent with MIEN1 having a potentially more robust connection to clinical prognoses and outcomes.

Strengths and limitations

Despite the consistency of the above correlative findings across several datasets, the approaches described above have several limitations. While previous research has supported direct functions of GRB7, ERBB2, and MIEN1 in tumor development, the analyses described here were not supported by evidence of molecular mechanisms or causation. Also, the observation that survival distinctions for these genes were not uniformly statistically significant across all datasets raises the question of whether this lack of uniformity is due to the coarseness of the data or approach or, in fact, to micro-CN distinctions limited to specific genes. In addition, while the correlation between gene CNs and RNA expression provided evidence that greater CNs of a gene do indeed represent greater RNA expression, there was no opportunity to further correlate the CNs with protein expression.

## Conclusions

In conclusion, the analyses presented provide new evidence, using a novel approach, that MIEN1 CN amplification is associated with poorer outcomes in both lung adenocarcinoma and cervical cancer. In certain cases, increased MIEN1 CNs were associated with decreased survival probability, whereas ERBB2 and GRB7 CN amplification were not. These results raise the questions of whether MIEN1 CNs should be further considered as a means of patient stratification and whether MIEN1 may be a useful target in the development of anticancer therapeutics, independently of ERBB2 therapeutic target considerations.

## Appendices

Supporting online materials are available at https://usf.box.com/s/y2pp6yl46g3vnlfah7fwccckjbohjo1a
